# Trends in SPR Cytometry: Advances in Label-Free Detection of Cell Parameters

**DOI:** 10.3390/bios8040102

**Published:** 2018-10-30

**Authors:** Richard B. M. Schasfoort, Fikri Abali, Ivan Stojanovic, Gestur Vidarsson, Leon W. M. M. Terstappen

**Affiliations:** 1 Medical Cell BioPhysics Group (MCBP), University of Twente, 7500 AE Enschede, The Netherlands; f.abali@utwente.nl (F.A.); ivanstojanovic172@msn.com (I.S.); l.w.m.m.terstappen@utwente.nl (L.W.M.M.T.); 2 Interfluidics BV, 7483 AL Haaksbergen, The Netherlands; 3 Department of Experimental Immunohematology, Sanquin Research and Landsteiner Laboratory, 1066 CX Amsterdam, The Netherlands; G.Vidarsson@sanquin.nl

**Keywords:** surface plasmon resonance, cytometry, SPR imaging, single-cell secretion

## Abstract

SPR cytometry entails the measurement of parameters from intact cells using the surface plasmon resonance (SPR) phenomenon. Specific real-time and label-free binding of living cells to sensor surfaces has been made possible through the availability of SPR imaging (SPRi) instruments and researchers have started to explore its potential in the last decade. Here we will discuss the mechanisms of detection and additionally describe the problems and issues of mammalian cells in SPR biosensing, both from our own experience and with information from the literature. Finally, we build on the knowledge and applications that has already materialized in this field to give a forecast of some exciting applications for SPRi cytometry.

## 1. Introduction

The common field where Surface Plasmon Resonance (SPR) sensing has been applied for almost three decades is to measure label-free biomolecular interactions in the vicinity (within ~300 nm) of the sensor surface in real time. Interactions of (bio)molecules with molecular weight between 1000 Dalton and 500 kilo Daltons is the traditional range for detection by SPR. With new technological advances in various niches of research and the availability of more than 25 SPR instruments and manufacturers [1], applications beyond traditional molecular binding experiments are entering the market. We observe not only a good competition in the traditional field but also new geometrical designs of the optical and fluidic parts suited for novel applications. The real-time imaging capabilities of this technique allow observation of dynamic changes at the surface. The sensor surfaces may be printed with multiple ligand molecules and the refractive index change caused by binding of the analyte can be applied for direct cellular-binding studies, observing physiological changes or for sensing of secreted proteins from single cells. In this review, recent studies involving analysis and detection of mammalian cells using SPR imaging are summarized and its future potential is highlighted [2,3,4]. Bacterial cell analysis, as reviewed in the paper of Abadian [5], is excluded because the typical features and special protocols for bacterial cell analysis are different with respect to mammalian cell protocols.

In some publications [6,7], it has been successfully shown that SPR can be used to give added value to cell analysis by measuring viable cells or the products of viable cells label-free in a multiplex manner [8]. These studies also underlined that SPR imaging cytometry, being a real-time, low-light-level, and label-free imaging technique, can be developed further in order to reveal its full potential and provide added value to cellular analysis [9]. The field of SPRi cytometry covers at least the following applications: (1) Direct detection of cell membrane antigens, morphology changes, and apoptosis; (2) ranking the affinity of cell surface antigens to antibodies; (3) detection of secreted molecules produced by single cells.

Below we will also try to explain the relevant mechanism for understanding the physical phenomena underlying cellular detection by SPR. In Section 1, the features of cells immobilized on a SPR sensor surface are summarized [10,11,12,13], including the responses to cellular morphology changes [14] and processes of apoptosis [15]. Additionally, it shows the potential for SPRi cytometry to measure the presence or absence of cell surface antigens on red blood cells (RBCs). Alternatively, SPRi cytometry is described for the ratio of the number of various cell membrane antigens [16]. In Section 3, we summarize a novel SPRi strategy that can be used to rank the avidity of ligands to cellular receptors or avidity of antibody-IgG-opsonized cells (red blood cells, RBCs) to IgG-Fc-receptors (FcγR). It also reveals the difficulty of getting the affinity constants for antibody binding to living cells. Finally, the SPRi cytometry field includes the monitoring of secretion of cellular products (e.g., antibodies) by living cells as described in Section 3.

For all these applications, one can argue why SPR was not applied earlier for monitoring cellular interactions. (A) For practical reasons, most commercial SPR instruments (e.g., BIAcore) are configured with optics on top of the fluidics to avoid leakage of liquid into the optical compartment of the instrument. In these instruments, cell sedimentation will occur at the surface opposite to that of the SPR sensor and cells that sediment are not detected. (B) The majority of SPR instruments use fluidic cartridges with tiny valves for operation and sample injection, which are prone to clogging when injecting a cell suspension. (C) Besides SPR imaging, the binding of the cell to a surface can be visualized with microscopic techniques and fluorescent staining so the need to apply costly SPR equipment for binding studies was not considered. (D) A cell is many times (>20x) larger than the penetration depth of the evanescent field (~0.5λ of the incident light) so only a small part of the cell is within the decaying evanescent sensing field. (E) Suspended cells under certain shear rate conditions are bounced from the sensor surface and will not interact because of the size of the cell in relation to the thickness of the stagnant layer of 1–5 µm [17] under laminar flow conditions. (F) Cells need to be resuspended homogeneously to ensure uniform coverage of the sensor surface (e.g., using back and forth flow before injection of the cells in the label-free sensing area). Many systems allow only one direction of flow. (G) Cell interactions to immobilized antibodies/antigens do not show 1:1 binding kinetics because of the multiple antigens present on a cell. Avidity-based models using serial dilutions of the ligand densities should be applied for the number of interactions and for qualifying the affinity of the cell antigen antibody interaction. (H) A living cell attached to the surface will not generate a stable baseline, which is a starting point for injection of an analyte that interacts with the cell. (I) Responses due to cell adhesion will depend on the orientation and packing density of bound cells. A nonlinear response is expected close to saturation levels of the surface area. (J) Last but not least: costs! Many tests need disposable SPR sensor chips that are 100 times higher in price than a conventional ELISA plate or a microscopic slide. Simple agglutination tests are alternative methods to measure cellular interactions. Additionally, the investment in capital-intensive equipment is a drawback. Commercial applications that could drive and intensify developments are not (yet) present.

In the next sections, two application areas of cellular interaction studies are highlighted and the features, benefits, and drawbacks are given. Our findings are based on a selection of papers published in literature since 2014 and our own experience with the observed physical phenomena. 

## 2. Direct Detection of Cell Membrane Antigens

Generally, SPRi detects refractive index changes within the evanescent field. Most cellular reactions involve a complex signaling cascade of biomolecular processes, which can contribute to local changes of refractive index in the cytosol affecting the overall SPR signal. This makes interpretation of specific SPR dynamics of cells problematic. To ensure reproducibility of SPR experiments, cells have to interact with the gold surface of the SPR sensor and form a tight monolayer. However, when a cell is exposed to different isotonic conditions, the cell volume and refractive index change [18]. The complex effects we observe are based on the premise that cellular activity induced by external agents is often associated with changes in cellular morphology, which in turn should lead to a variation of the effective refractive index at the interface [19,20]. Moreover, when these reactions happen simultaneously, refractive index changes are attributed to more than one underlying process [21]. In this case, signals can become impossible to interpret and cannot be linked to specific cellular reactions [22]. For this reason, cell assays must be designed so that SPR response can be attributed to a specific reaction of interest of the cell [23,24,25,26]. An elegant way to deconvolute the responses from different processes is by applying at least two wavelengths for tuning the penetration depth of the evanescent field [27]. It enables optimization for exploring cellular structures and secretions. In theory, two SPR strategies for SPR cytometry exist: (1) Cells are injected as analytes and bind to specific ligands (e.g., antibodies) immobilized to the sensor surface. Only the cell part that is within the evanescent field volume (~300 nm) will be detected. (2) Cells are immobilized to the sensor surface and act as ligands for the analytes that are injected to observe the SPR response. In this case, cells can be immobilized by first injecting the cells as analytes, followed by sedimentation. Only the “ligand” part of the cell that is within ~300 nm of the sensor area is analyte-responsive.

Label-free detection of the interaction between specific receptors on the cell surface and their ligands using SPRi would have distinct technical advantages compared to traditional cell-based analysis techniques such as flow cytometry or fluorescence microscopy. Antibodies used in SPRi do not need to be conjugated, eliminating possible influence on the antibody affinity due to conjugation. However, instead of conjugation, the antibody should be immobilized to the sensor surface. Studies focusing on retrieving qualitative and quantitative information on cell membrane antigens are relevant for various applications [28]. Multiplexing can be achieved by covalent coupling of the various antibodies in spots on the surface of an SPRi sensor.

**Figure 1:**
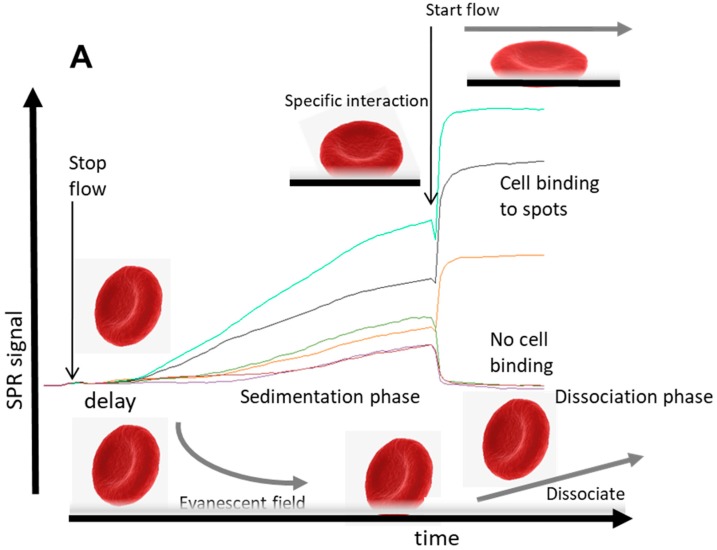

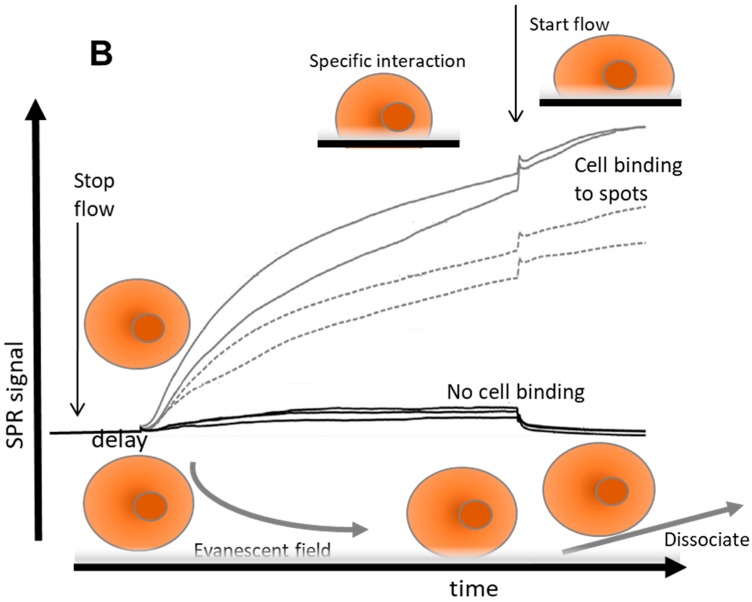
Principles of cellular SPR. (**A**) For nonadherent and rigid cells such as RBC and (**B**) for adherent cells. Generally, mammalian cells sediment and penetrate into the evanescent field and will be detected. The cell surface antigens will bind to specific ligands on the surface and will deform the cell. The signal depends on anchoring points of ligands with the cell. More anchoring points of ligand–cell surface antigens interactions allow that the cell penetrates deeper in the hydrogel of the sensor surface giving rise to higher SPR signals. Flow induces a force to cells and effectively deforms the cell and a larger part of the cell will be detected. This is reversible for rigid cells such as RBC in (**A**), but can lead to spreading in time and during exposure of the cells, a continuously increasing SPR signal occurs in (**B**) [30].

**Figure 2:**
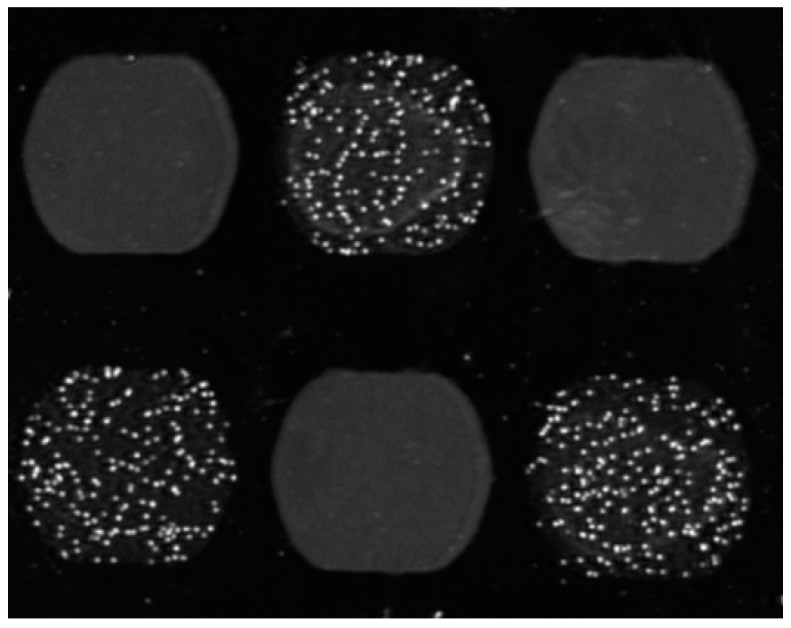
Cells imaged on an SPR sensor. T-lymphocytes are visible in the SPR image on a checkerboard of anti-CD3 spots (with speckles) and BSA spots (grey areas). The image has been taken after the flow started and specific cell binding is observed on anti-CD3 spots and the not-bound cells on BSA spots were washed away. The IBIS MX96 instrument (IBIS Technologies, Enschede, the Netherlands) was used. It contains reversed optics and back and forth flow fluidics, allowing for sedimentation and controlled flow mixing of cells. Valveless injection of samples and wide 1 mm (internal diameter) tubing allows smooth aspiration of cell suspensions without clogging.

This section describes an SPR imaging detection method for typing red blood cells (RBCs) [29] using a critical sedimentation step followed by a wash step of antibody spots recognizing RBC antigens on a sensor surface. The method for antigen typing of red blood cells can in principle be extrapolated to any cellular detection as shown in Figure 1 and for T-cells in Figure 2.

**Figure 3:**
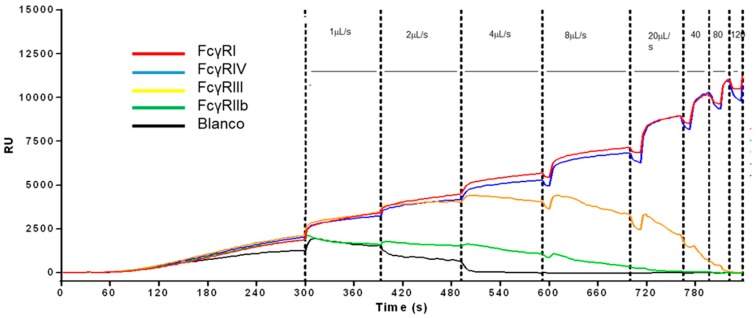
Affinity profiling using SPR. T/S sensorgram for label-free cell profiling measured in the IBIS MX96 (unpublished results). Red is FcγRI, blue is FcγRIV, orange is FcγRIII, green is FcγRIIb (all mouse), and black is blanco. Sedimented cells are subjected to an incremental increase in flow rate. When the drag forces of flow are too high, the cells will dissociate from the surface. RBCs will bind or dissociate dependent on the affinity and number of receptors (avidity). Increasing flow rate, stepwise (indicated by vertical dotted lines) from 1mL/s (at 300 s) up to 120mL/s, allows determining how well the cells bind to the spotted FcγRs. Avidity was determined by measuring the binding strength of mouse IgG2a-opsonized RBCs to c-terminally biotin tagged FcγR on a streptavidin sensor measuring binding to all mouse FcγR simultaneously for each antibody (n = 6).

From these observations, the kinetic process of cell binding has at least two typical features: first, after injecting the cells, a delay of the response signal is observed after stopping the flow. This typical delay occurs when large particles (cells) are applied. Second, cells that bind specifically to the immobilized ligand molecules will show an upward response after starting the flow again, while a downward response is observed from reference spots or other spots that do not bind cells. If the shear rate is below a certain critical value of disruption, the response is stable and the cells will stay on the spots and no off-rate is observed. These typical effects can be explained as follows: (1) In flow, mammalian cells are transported to the sensor surface in laminar flow with the highest flow in the middle of the channel and nearly zero flow close to the wall (so-called stagnant layer [17]). After a lateral transport of the cell suspension over the sensor surface, cells will not be in direct contact with the sensor surface because of shear forces, but will stay in the middle of the flow. Therefore, the mammalian cells will only enter the cell-free stagnant layer once the flow is stopped, allowing settling into the evanescent field of the sensor, and hence the delay of sedimentation response is observed. (2) After reinstating the flow, sedimented cells which are not recognized by the specific interactions with the sensor will be washed away of the evanescent field and the response will return to the baseline (lower right in Figure 1A). In contrast, when cells are bound to specific spots of the sensor surface (e.g., through antibody–antigen interactions), the flow will induce a reorientation, repacking, or pressing down of the cells. This is particularly true for biconcave RBC and rigid, increasing the measured effect (Figure 1A, top right). Energetically, it is beneficial that cells are pressed closer to the surface induced by the flow, because the flow forces the anchored cells outwards towards the stagnant layer. This results in a larger part of the specifically bound cells that is localized in the evanescent field and an upward response is observed. However, when the flow is very high with relative low affinity/avidity to ligands on the sensor, the shear forces on the cells can overcome the strength forcing the binding to the surface and they will be released (data shown in Figure 3). Hence, discrimination can be made between weak/nonbinding cells compared to specific bound cells by adjusting the flow speed after the sedimentation phase. This observation led to a strategy to qualify avidity of cell interactions that correlate with the number of anchor points.

Using this SPR scheme and method, a label-free cell profiling of RBCs has been developed further, allowing for simultaneous typing of all major blood groups. RBCs were injected in a flow chamber on top of a spotted sensor surface. Spots contained antibodies to various RBC membrane antigens. The full analysis cycle for RBC profiling was less than 6 min. The sensor surface could be regenerated at least 100 times, allowing the label-free determination of the cell surface antigen profile of RBC (Schasfoort et al. and Szittner transfusion 2018 resubmitted).

The presence and absence of RBC surface antigens can be measured but the question arises: can we can quantify the number of surface antigens too? The behavior of an RBC or a (non)adherent mammalian cell is slightly different. Adherent cell and platelets show, both in flow and nonflow conditions, a continuous increasing signal [31], while the RBC signal is stable under flow conditions. Nevertheless, data with adherent cells still yields quantitative information, and can be mixed on an SPRi array to simultaneous label-free detection of 44 antigens on cells within 20 min [16]. A higher SPR response can mean that more cells are captured [32] but also gives information about the antigen expression on the cell surface as this correlates with flow cytometry measurements. An explanation is that cells with high abundant surface markers will insert and spread faster and deeper in the gel layer, while cells without interaction points will do this slower and penetrate the hydrogel layer less easily and therefore be less visible in the evanescent field [33]. A drawback using adherent cells is that the experiment still cannot be repeated with the same sensor, although regeneration of platelets is possible with mild detergents and low pH [34]. Though regeneration of sensors with bound RBCs proved possible, regeneration conditions for other cells and immobilized ligands were not (yet) found and more work should be carried out to find protocols for repeated injections of various cell lines on clean and regenerated sensor surfaces.

## 3. Ranking the Affinity of Cell Surface Antigens to Antibodies

Recently, a new approach for determining the affinity of cell receptor antibodies has been applied. This work showed that it is possible to rank the avidity of cell interactions (e.g., different IgG subclasses) for their binding to various cell surface antigens (Fc-receptors). The method is very similar to typing of RBC [29,35] but instead of having antibodies on the sensors, the sensor spots were equipped with different IgG-Fc receptors (FcγR). RBCs opsonized with different antibodies were then ranked for their differential avidity for each FcγR simultaneously (Figure 3). The same methods can also be used to affinity-rank antibodies against difficult targets (e.g., against new therapeutic antigens that can only be expressed on cells). Direct detection of the antibody that binds to a sedimented cell line was not possible because of highly unstable baselines due to activity of the cells that overrules the expected antibody association and dissociation responses. However, we found that the release or dissociation of cells from the sensor surface depends on several factors (e.g., the flow velocity, the number of antigens on the cell, the amount of antibodies used for opsonization, the affinity of the antibodies for antigen, and affinity of the IgG to the receptor, but also the FcγR-density on the sensor). When a ligand gradient (in this case, IgG-opsonized RBC) is applied in combination with increasing flow rates (shear rate), then ranking the affinity could possibly be measured on multiple receptor–Ab combinations, allowing ranking based on the shear force (flow rate). The shear on cells depends on the local velocity profile of the buffer stream on the immobilized cells. Figure 3 shows the feasibility that the ligand density of the immobilized antibodies and the velocity of the buffer flow is important to cell receptor affinity ranking.

By applying a uniform force on the cells, a ligand density series of antimembrane antigens will tune the position where cells at a certain velocity will dissociate from the spots. In this way, affinities of receptors on cells can be compared and ranked to each other when simultaneously different antibodies are immobilized in a ligand density series. The ranking can be achieved when similar conditions of functional ligand densities can be created. This method can only be applied for affinities in the µM range. For affinities in the nM range, including highly abundant cell surface receptors, it is unlikely that a still-intact living cell can be dissociated from the sensor surface for ranking the affinity. However, an nM affinity ranking can possibly be circumvented by applying unfavorable buffer conditions in addition to a low level of receptor density and/or low level of opsonization.

## 4. Quantifying the Secretion of Cells

As SPRi know-how and microfluidics evolved the last decade, various studies have reported the use of SPRi to study cellular responses and interactions, summarized in the review by Abadian [5,36]. In the paper of Milgram [37], SPR cytometry studies were performed with cell lines on a microarray of various antibody ligands. It describes in detail surface chemistries of coupling ligands for cell binding, cell secretions, and cell regeneration studies from the SPR sensor surface using a Horiba Scientific SPR imager. From these studies, we postulated that it should be possible to measure also the products secreted by cells on a single-cell level by SPRi.

**Figure 4:**
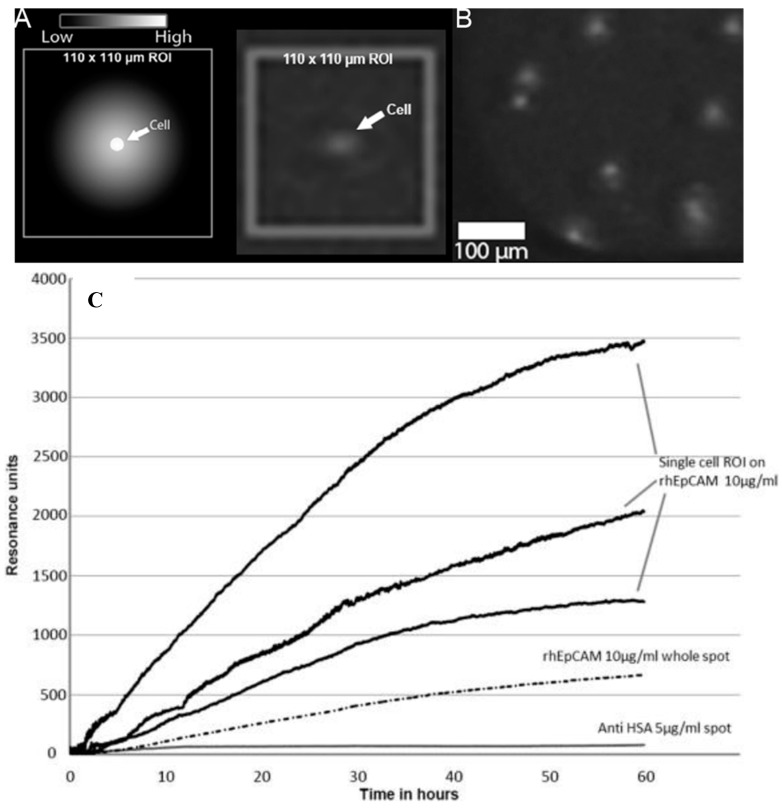
Single-cell antibody secretion monitored with SPRi. Panel (**A**): Schematic representation of cell that secretes antibodies; the sensor surface captures the secreted molecules in a halo around the cell. Panel (**B**): SPR-image of VU1D9 cells on top of the sensor surface with secreted anti-EPCAM antibodies. Panel (**C**): SPR sensorgram showing the variation of single-cell production of anti-EpCAM by VU1D9 cells. Noise levels are high due to very small, 110 µm^2^ sized Regions of Interest. As a reference, anti-HSA and rhEpCAM whole spot averaged signals with large Region of Interests are shown. The signals were zeroed in order to compensate for the background.

Traditionally, detection and quantification of single-cell secretion is a complicated procedure [38,39]. To investigate if it is possible to measure the secreted products from a single cell, an IBIS MX96 SPR imager was modified so that single cellular production could be monitored for prolonged periods of time at 37 °C [40]. (See Figure 4.)

As a model system, a hybridoma cell line producing VU1D9, an IgG1 monoclonal antibody (of murine origin) recognizing EpCAM, was used. In an additional paper [41], a mathematical model was developed that showed that 99.1% of the produced antibodies of a single VU1D9 cell (production is ~ 100 molecules per cell per second) will bind directly to the sensor surface and only 0.9% was lost due to diffusion into the bulk. Therefore, it can be stated that the capacity of the sensor surface is sufficient to capture the molecules produced by single cells. So, SPRi can be used as an accurate quantification method for single-cell protein production [42].

**Figure 5:**
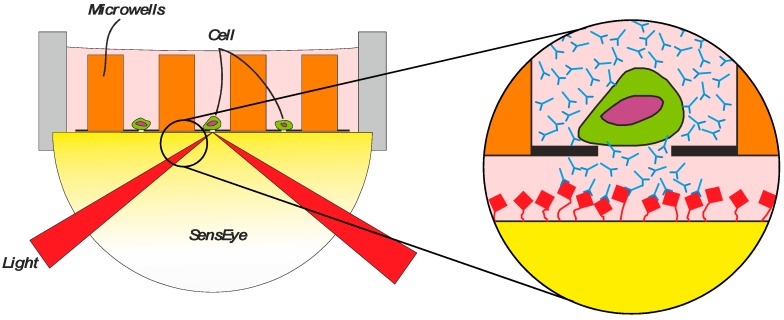
The principle of the McSPRinter. The microwell arrays with single cells is attached to a SPR sensor surface and immersed in cultivation medium. The product of the cells diffuses via the 1 µm membrane to the sensor surface. Reprinted from F. Abali et al. [44] Copyright 2017 with permission from Elsevier.

This method can be further improved using McSPRinter technology (Microwell cell Selection PRinter) (Figure 5) to seed single cells in individual wells [43] (VyCAP, Deventer, The Netherlands). The self-seeding microwell chip with an array of 80 × 80 wells was combined with SPRi to monitor, track, and quantify the secretion of antibodies from each of the individual cells in the wells. Selection of cells from a pool of thousands of cells can be carried out in just two hours of incubation.

The protocol for selecting single cells based on criteria as the quality and quantity of the secreted product is according to the procedure described by Abali et al. [44]:

(1) A cell suspension is transferred to a microwell chip containing an array of microwells and by a small vacuum, a cell suspension flow drags a single cell into a pore of 5 μm in the bottom of the microwell. (2) The cell blocks the pore and the remaining fluid and cells will be diverted to the next available microwell, resulting in a fast distribution of single cells in individual microwells. (3) After this seeding process, the microwell device is connected to a Surface Plasmon Resonance imaging sensor (SensEye^®^, Ssens BV, Enschede, The Netherlands) with a specific selection surface. The SensEye® sensor and microwell filled with single cells are incubated for a certain period of time, allowing the cells to secrete specific molecules which will be captured by ligands immobilized on the sensor surface. (4) In this way, a transfer of specific molecules as individual cell product (e.g., antibodies from CHO) is captured by the sensor surface and the production rate of these molecules can be monitored for each cell individually label-free and in real time. Next, the microwell array is removed from the sensor surface and the sensor is inserted again in an SPRi instrument (IBIS MX96, IBIS Technologies, Enschede, The Netherlands) for exposing the microwell arrayed sensor surface to a specific biomolecular interaction. The amount of specific product, but also the affinity criteria as on and off rate for each secreted molecule can be determined. The criteria for selecting interesting cells are set for each position of the array and the coordinates of the cells of interest in the microwells are determined. The single cells are isolated from the microwells by punching the selected cells out of the microwell array into a culture dish for clonal expansion.

This technology opens an avenue to measure the kinetics of products secreted by single cells and harvesting the cells of interest, thereby increasing our understanding of cellular processes. One can envision the simultaneous measurement of a variety of products secreted by cells using SPRi, such as specific proteins, hormones, neurotransmitters, cytokines, and exosomes.

## 5. Conclusions

In the last five years, SPRi cytometry has been recognized as a new field of research grouped in three main areas. In this review, the mechanisms of detection are described, supported with some examples from the literature. 

The first group is the measurement of the presence of cell membrane antigens and ratio of various cell membrane antigens by injecting cells as an analyte. The detection protocol is: injection of the cells, stopping the flow enabling sedimentation, followed by starting the flow to observe specific cellular interactions. It includes the determination of the affinity of biomolecules (e.g., antibodies) to cell surface membrane antigens. For the first time, a feasible strategy is shown to rank the weak avidity of cells to corresponding IgG-opsonized cells to FcγR. However, it remains technically difficult to rank high affinity binders to cell receptors. 

When cells are applied as ligand, so the analyte triggers an event, complex mechanisms can be monitored (e.g., morphological changes, apoptosis due to external triggers). This is the less favorable way of operating SPR cytometry due to the complexity of the signals. Living cells may deform, spread, penetrate in the hydrogel, anchor, swell, or shrink, and division of the cell in two cells and even migration can be observed. All these effects result in local refractive index changes and the baseline of the sensorgram fluctuates heavily when experiments are carried out at 37 °C. 

Additionally, SPRi cytometry field can be applied to the monitoring of cellular secretion of molecules by living cells. The Microwell cell Secretion PRinter (McSPRinter) technology enables not only the monitoring of the single cellular secretion of thousands of cells simultaneously, but also it makes possible to isolate the individual cells of interest with microwell MEMS technology. An advantage of this technology is that the cell is not in direct contact with the sensor surface but only the secreted molecules. 

Although SPRi cytometry is still in its infancy, we expect that definitely some unique applications, such as multiplex cell surface antigen profiling and a promising real-time, single-cell secretion detection, will appear in the market in the coming years.
